# Recommended dose voxel size and statistical uncertainty parameters for precision of Monte Carlo dose calculation in stereotactic radiotherapy

**DOI:** 10.1002/acm2.13077

**Published:** 2020-10-30

**Authors:** Simon K. Goodall, Martin A. Ebert

**Affiliations:** ^1^ School of Physics, Mathematics, and Computing Faculty of Engineering and Mathematical Sciences University of Western Australia Crawley WA Australia; ^2^ GenesisCare Wembley WA Australia; ^3^ Department of Radiation Oncology Sir Charles Gardiner Hospital Nedlands WA Australia; ^4^ 5D Clinics Perth WA Australia

**Keywords:** Monte Carlo, stereotactic radiotherapy, treatment planning

## Abstract

Monte Carlo (MC)‐based treatment planning requires a choice of dose voxel size (DVS) and statistical uncertainty (SU). These parameters effect both the precision of displayed dose distribution and time taken to complete a calculation. For efficient, accurate, and precise treatment planning in a clinical setting, optimal values should be selected. In this investigation, 30 volumetric modulated arc therapy (VMAT) stereotactic radiotherapy (SRT) treatment plans, 10 brain, 10 lung, and 10 spine were calculated in the Monaco 5.11.02 treatment planning system (TPS). Each plan was calculated with a DVS of 0.1 and 0.2 cm using SU values of 0.50%, 0.75%, 1.00%, 1.50%, and 2.00%, along with a ground truth calculation using a DVS of 0.1 cm and SU of 0.15%. The variance at each relative dose level was calculated for all SU settings to assess their relationship. The variation from the ground truth calculation for each DVS and SU combination was determined for a range of DVH metrics and plan quality indices along with the time taken to complete the calculations. Finally, the effect of defining the maximum dose using a volume of 0.035 cc was compared to 0.100 cc when considering DVS and SU settings. Changes in the DVS produced greater variations from the ground truth calculation than changes in SU across the values tested. Plan quality metrics and mean dose values showed less sensitivity to changes in SU than DVH metrics. From this study, it was concluded that while maintaining an average calculation time of <10 min, 75% of plans could be calculated with variations of <2.0% from their ground truth values when using an SU setting of 1.50% and a DVS of 0.1 cm in the case of brain or spine plans, and a 0.2 cm DVS in the case of lung plans.

## 1 INTRODUCTION

Monte Carlo (MC) algorithms are widely considered to be the gold standard for the calculation of dose distributions for radiotherapy treatments.^1^, ^2^, ^3^ Treatment planning systems (TPSs), which implement such MC algorithms, have become commonplace in modern radiotherapy due to the time savings introduced by advances in computing processors and the optimization of codes specifically for treatment planning.^1^, ^4^ These optimizations have seen the implementation of virtual source models, transmission filters, and photon cut‐off energies along with variance reduction techniques such as interaction forcing, electron history repetition, and Russian Roulette, all of which are used within Monaco (Elekta, Stockholm, Sweden) to achieve acceptable calculation times.^1^, ^5^, ^6^, ^7^, ^8^ The choice of different user settings, however, such as the dose voxel size (DVS) and statistical uncertainty (SU), still play a large role in the time required to complete a calculation.

When completing an MC‐based calculation, the dose distribution region is divided into voxels of equal volume, defining the spatial resolution. The dose deposited within a given voxel is represented at a single location before an interpolation between voxels is completed to display a complete distribution.^6^ The SU setting is unique to MC‐based algorithms and controls the level of statistical noise remaining within the final calculation. As the SU value is decreased, the number of simulated histories is increased, resulting in a lower level of statistical noise present in the final display.^6^

For a single beam, the dose displayed at a given point, *i*, in a dose distribution calculated with a given SU, $D_{i}^{\mathrm{SU}}$, can be assumed to be a summation of a theoretical noise‐free, or 0% SU, dose, $D_{i}^{0}$, and an error sampled at random from a Gaussian distribution with a mean of 0, *G(σ)*, and a variance, *σ*, proportional to the square root of the dose in that voxel^1^, ^9^, ^10^, ^11^

(1) $$D_{i}^{\mathrm{SU}}=D_{i}^{0}+G\left(\sigma_{\mathrm{Dmax}}\sqrt{\frac{D_{i}}{D_{\max}}}\right)$$

where $\sigma_{\mathrm{Dmax}}$ is the variance in the voxel with the maximum dose, $D_{\max}$. A specification of $\sigma_{\mathrm{Dmax}}$ typically constitutes the user‐definition of SU.

A number of studies have investigated the effects of varying the SU on the ability to assess the suitability of a treatment plan in a theoretical fashion using MC codes not customized for treatment planning in clinical practice.^3^, ^10^, ^11^ Others have looked at the possibility of removing the noise completely.^12^, ^13^, ^14^, ^15^ Although these studies can suggest SU settings, which may be appropriate, it can be hard to understand or quantify the direct effects they have on a clinical distribution due to their limited examples or purely theoretical nature. In addition, the suggested SU settings are made at the discretion of the authors, they may not be applicable to all treatment techniques or clinical requirements and they may not directly translate to commercial systems using variance reduction techniques.

Treatments requiring the highest levels of accuracy, such as stereotactic radiotherapy (SRT), which uses small fields and steep dose gradients to allow dose escalation to small targets, stand to benefit strongly from using more accurate dose calculation algorithms.^1^, ^4^, ^16^ In particular, areas of rapid change in density, such as the lung and spine (bone), are expected to show significant improvements in accuracy when calculated using MC algorithms.^11^, ^16^ The complexity of SRT treatment plans have been shown to require a DVS of not >2 mm in any direction to ensure the dosimetry is accurately represented.^16^, ^17^, ^18^ To the knowledge of the authors, no previous studies have investigated directly, or quantified, the effects of the SU settings used in commercial TPS upon SRT dose distributions and their assessment of fitness for clinical use.

Cumulative dose volume histograms (DVHs), or a sample of key metrics derived from them, are often used to evaluate a treatment plan along with calculated plan quality indices. DVHs display the dose along the horizontal axis and the relative volume of a structure, receiving the dose, on the vertical axis. For conventional radiotherapy plans, an ideal target DVH curve therefore represents a step function with 100% of the target receiving the prescription dose exactly. It has been shown that target DVH curves, which approach such a step function, display greater variation with changing SU than organ at risk (OAR) curves which generally have shallower gradients.^11^

Stereotactic radiotherapy treatment plans could be expected to differ in their response to changes in SU, more so than for conventionally fractionated radiotherapy. Typical SRT plans display highly inhomogeneous dose distributions across target volumes with maximum doses in excess of ~130% of the prescription dose, compared to ~107% for conventional radiotherapy.^4^, ^16^ Consequently, the prescription isodose may be a low as ~70% of $D_{\max}$ within an SRT plan compared to ~90% for conventional radiotherapy. As a result, the target DVH curves for SRT treatment plans do not approach a step function. The gross target volume (GTV) and planning target volume (PTV) are also typically small and hence contain only a relatively small number of voxels.^4^ Large random fluctuations in calculated dose due to statistical noise could therefore have a larger effect on plan metrics, even if they occur only for a few voxels.^1^, ^6^

When deciding upon optimal DVS and SU settings, it is also important to consider the effect on the calculation times, which if too large can prevent practicable clinical implementation. Changes in DVS are volumetric, as such that the calculation time $T_{c}$ is inversely proportional to the DVS setting cubed.

(2) $$T_{c}\alpha \frac{1}{DVS^{3}}$$

Reductions in the SU require additional particle histories to be considered such that for a fixed DVS setting

(3) $$SU\alpha \frac{1}{\sqrt{N}}$$

where *N* is the number of histories used^1^, ^6^, ^11^. It should be noted that Eq. (3) may not always be directly applicable for treatment plans which use multiple beams or control points (beamlets), which may not all contribute to calculation at the $D_{\max}$ voxel; however, it is a reasonable approximation even in these cases.^6^, ^10^

Combining Eqs. (2) and (3), one can therefore write

(4) $$T_{c}\alpha \frac{SU^{2}}{DVS^{3}}$$

when assuming all particle histories require the same time for simulation. This approximation can be used to show that for equal order variations of DVS and SU, DVS has a larger effect on the total calculation time. In addition, it can allow estimations of equal calculation times for given DVS and SU combinations.

This investigation aimed to determine the general applicability of Eq. (1) to complex volumetric modulated arc therapy (VMAT) dose distributions produced by the commercially available Monaco 5.11.02 TPS, and assess the relationship between the user‐defined setting of SU and $\sigma_{\mathrm{Dmax}}$. Following this, the investigation aimed to quantify the changes in key DVH parameters and plan metrics introduced when changing the SU setting for a range of SRT treatment plans to allow clinical teams to determine the level of uncertainty that would be acceptable within their practice. Finally, the investigation aimed to recommend clinically practicable values of DVS and SU for SRT treatment planning within the Monaco TPS.

## 2 MATERIALS AND METHODS

A total of 30 previously treated VMAT SRT treatment plans developed and delivered at GenesisCare using the Monaco 5.11.02 TPS were considered in this investigation. The plan cohort consisted of 10 brain, 10 lung, and 10 spine (bone) treatment plans. Cases were planned with one of two beam models with an energy of 6 MV or flattening filter free 6 MV (6FFF).

### 2.A Monaco

Upon setting a value of SU for a given plan, the Monaco 5.11.02 TPS considered in this investigation determines the number of particle histories required for the calculation using an empirical formula assuming all control points to have an equal weight. Once the simulation of these particles is complete, the achieved SU is calculated and additional histories are simulated if required, or the simulation is ceased.^6^ The SU can be set per plan or per control point, corresponding to the SU of the total dose distribution, or the dose distribution from a given control point, respectively. Within this investigation, SU per plan will be referred to unless explicitly stated otherwise.

For a given patient and plan combination, Monaco initiates a MC calculation from a fixed initial seed point. As such, the calculation of a plan completed with a low SU setting contains the same initial histories as the calculation of the plan with a high SU setting, combined with further additional histories. This implementation of a fixed initial seed point ensures that multiple repeat calculations using the same SU and DVS settings yield the exact same result. This is ideal for TPS quality assurance but prevents an assessment of the SU setting using the same plan and patient combination by limiting the sample size at each combination of settings to one.

The DVS is defined in Monaco by the “Grid Spacing” setting. This is a single value which results in the generation of equal volume, cubic, isotropic voxels. The minimum allowable setting is 0.10 cm and the resolution for incremental increases from this value is 0.01 cm.

### 2.B Ground truth calculation

Each plan was calculated with DVS and SU settings of 0.1 cm and 0.15%, respectively. The plan was then normalized to ensure that 98% of the PTV were covered by the prescription dose for brain or lung plans, and 90% of the PTV for spine plans as per clinical protocols. Throughout this investigation, for a given plan, the dose distribution calculated using these settings was considered the ground truth distribution of the TPS. These distributions represent the most precise calculations completed in this study; however, it is important to note that inaccuracies in the calculation may still be present. This assumption that a low SU calculation can be treated as noise‐free has been made in other publications.^10^, ^19^

### 2.C Investigated calculations

For comparison to the ground truth, each plan was recalculated using both a 0.1 and 0.2 cm DVS with SU settings of 0.50%, 0.75%, 1.00%, 1.50%, and 2.00% per plan. A total of 11 calculations per plan were therefore completed giving a total of 330 calculated dose distributions (including ground truths). The comparison of these dose distributions to the ground truth distribution was made to assess the effects of changing precision on the final displayed distribution. The underlying accuracy of the beam models and TPS must still be quantified by the clinical physicist.

### 2.D Assessment of the SU setting

To determine the applicability of Eq. (1) for the commercial TPS and VMAT, the variance of the Gaussian was estimated at intervals of relative dose level. All dose distributions were exported in DICOM format and the following calculations were completed in MATLAB R2015b (Mathworks, Natick MA).

For a given plan, the difference in each voxel dose, as a percentage of the ground truth calculation dose, $D_{i}^{0.15}$, was calculated

(5) $$\Delta D_{i}^{\mathrm{SU}}=100*\left(\frac{D_{i}^{\mathrm{SU}}‐D_{i}^{0.15}}{D_{i}^{0.15}}\right)$$

Each element of $\Delta D_{i}^{\mathrm{SU}}$ was then binned according to the value of $D_{i}^{0.15}$ as a percentage of $D_{\max}^{0.15}$ at the given location, *i*. A total of 100 intervals were created each with equal 1% width. This process was repeated for all plans considered in this investigation calculated with a 0.1 cm DVS relative to their respective ground truth.

The variance of the combined values of $\Delta D_{i}^{\mathrm{SU}}$ within a given relative dose interval, across all plans, was calculated to give a single value of $\sigma_{\mathrm{int}}$, as an estimate of the variance of the random errors for the given dose interval.

### 2.E Assessing DVH metrics

The value for a given DVH metric was recorded from each calculation of the treatment plan and compared to the ground truth for the given plan. The following DVH metrics were assessed during this investigation:

- Plan maximum dose
- GTV mean dose
- PTV coverage
- Spinal cord maximum dose
- Spinal cord PRV maximum dose

The PTV within this investigation was created via an isotropic growth from the GTV. For brain plans, the growth margin was 1 mm, for spine 2 mm, and for lung 5 mm. For spine plans, the planning risk volume (PRV) was a 2 mm isotropic growth of the spinal cord contour. When analyzing the variations in cord maximum dose, variations in the spinal cord and spinal cord PRV were considered as two separate points taken from the same calculation. This resulted in a total of 20 data points per SU and DVS combination, two from each plan.

The ability to precisely determine these metrics allows comparison of clinical outcomes across practices and informs clinicians on the likely clinical outcomes. ICRU report 91 recommends the near maximum, near minimum dose, and median dose of the target volume are recorded for level 2 reporting.^4^ The near minimum and median dose provide descriptions of the dose that the target will receive during treatment, allowing the clinician to advise on the probability of local control, based on similar patient outcomes. High maximum doses are often desirable in SRT due to the ablative intent of the treatment; however, precise calculation of the maximum dose is required to accurately report the prescription dose if defined as an isodose line relative to the maximum dose.^4^ When producing SRT plans for spinal treatments, the maximum spinal cord dose is often the limiting factor. If an excessive dose is delivered to the spinal cord, there are serious risks to the patient including myelopathy and a compromise of the PTV coverage may therefore be required.^20^, ^21^, ^22^

### 2.F Assessing plan quality indices

Two plan quality metrics commonly considered in SRT treatment planning were also investigated for every plan. The first was the Paddick conformity index (PCI) calculated as:

(6) $$PCI=\frac{TV_{\mathrm{PIV}}^{2}}{\left(TVxV_{\mathrm{RI}}\right)}$$

where $TV_{\mathrm{PIV}}$ is the target volume covered by the prescription isodose line, TV is the target volume, and $V_{\mathrm{RI}}$ is the volume of the prescription isodose. This index was originally developed to evaluate conformity for brain radiosurgery treatment plans but has value in all treatment planning.^4^, ^23^ The second was the gradient index (GI)

(7) $$GI=\frac{V_{R50}}{V_{\mathrm{RI}}}$$

where $V_{R50}$ is the volume of the isodose line equal to 50% of the prescription dose. For dose‐escalated SRT treatments, the gradient of the dose fall off outside of the treatment volume is important and can be assessed using this metric.^4^, ^16^ Although this value was also primarily introduced in the context of stereotactic brain treatment planning, it can provide guidance on plan quality, particularly if compared to local center baselines. It is included here for all plans for these reasons in addition to its ability to allow consideration of the variation of the 50% isodose line compared to the prescription (100%) isodose line when changing the SU.

### 2.G Treatment plan calculation time

The Monaco optimization console was used to determine the time between calculation initiation and the final control point completion. All calculations were completed using the GenesisCare clinical TPS servers with Intel® Xeon® CPU E5‐2690 v4 processors @ 2.60 GHz with 14 Cores and 128 GB of random access memory (RAM). Assignment to a specific server was via Citrix to the sever with lowest use at the time of opening the Monaco application. Each server was limited to a maximum of two users at any time; however, no record was kept of the number of users on the server while the calculations were completed. The only exception to this is the calculation of the ground truth dose distributions which were calculated overnight and the time for these calculations is therefore not reported.

## 3 RESULTS AND DISCUSSION

### 3.A Assessment of the SU setting

**Fig. 1:**
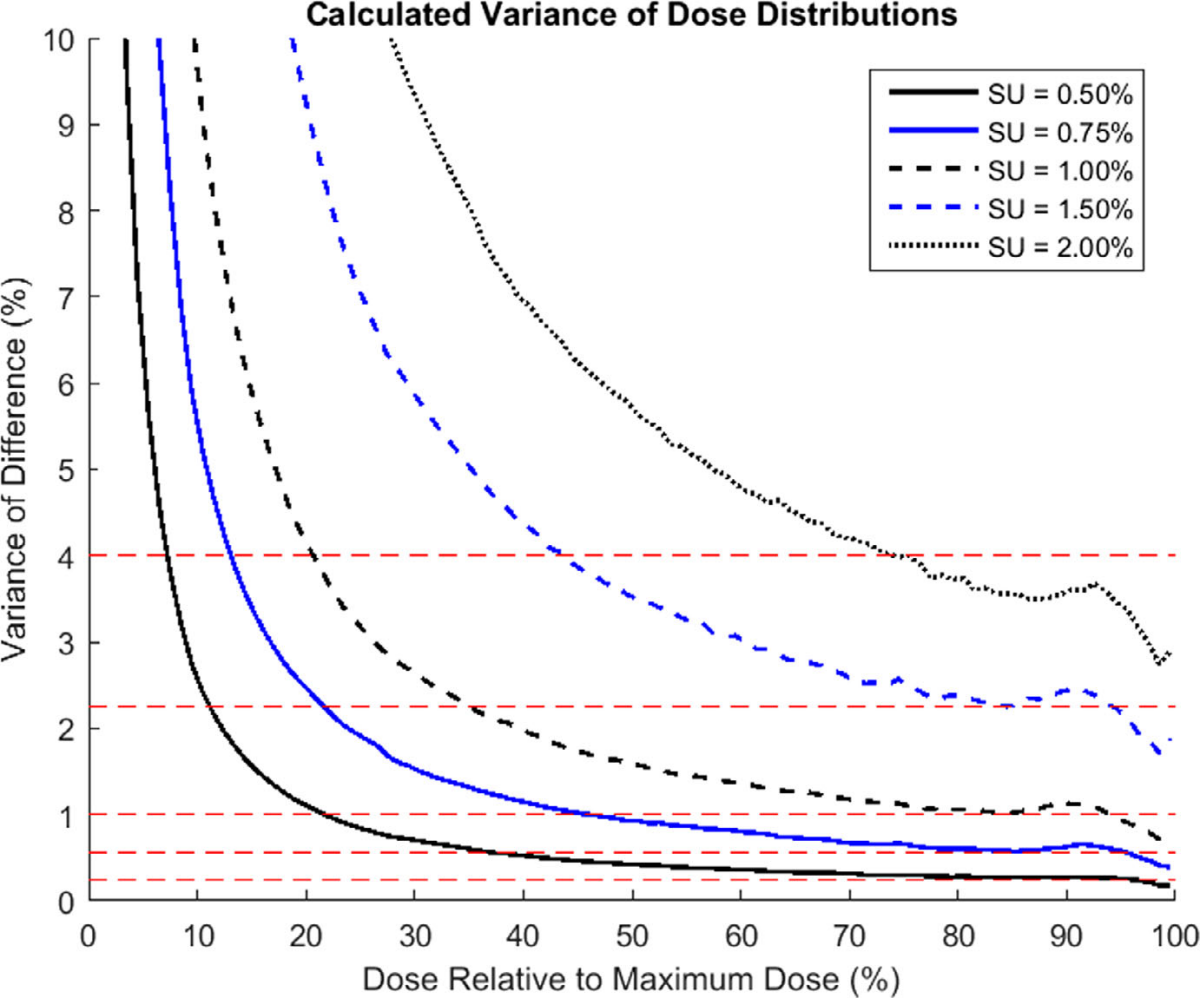
Calculated variance of the differences between the ground truth and a calculation with a given statistical uncertainty (SU) for all plans combined versus the relative dose of the interval of calculation. Each calculated variance is plotted at the mid‐point of the interval of consideration. The red dashed lines indicate the values of SU^2^ for the SU settings investigated.

The variance of the dose within a given interval relative to $D_{\max}$ for all plans considered in this investigation is shown in Fig. 1 for each setting of SU.

The calculated variance was shown to be a minimum at the maximum dose and subsequently increase for lower dose levels. At low dose values, an asymptote is observed in the calculated variance due to the calculation relative to the local dose. Around the maximum dose, the calculated variance is seen to be lower than the set SU value for all settings. From Fig. 1, there are two points of interest which may be inferred about the setting of SU within Monaco. Firstly, the SU value, referred to within the Monaco TPS as “Statistical Uncertainty”, appears to be representative of the standard deviation rather than the variance of the statistical noise. Secondly, for settings of SU ≥ 1.00%, the variance in the calculated dose differences is significantly below those expected from Eq. (1).

The reduction in calculated SU compared to expected is likely due to the predictive nature of the Monaco TPS in determining the number of histories required to achieve a given SU. For larger SU settings, the number of histories can be initially overestimated resulting in a lower than requested SU following the completion of the simulation. Following personal correspondence with Elekta, it was also identified that if the number of histories required by the prediction corresponds to a per control point SU of >12%, the number of histories will be increased until an SU of 12% per control point is predicted, resulting in a final calculation with a lower SU per plan than requested in the calculation settings. This is in agreement with initial findings within this investigation during which plans were recalculated with a SU setting of 3.00%. This step was abandoned when the calculated dose distributions were shown to be identical to those completed with a SU setting of 2.00%. The SU setting which corresponds to 12% per control point is dependent upon the plan, number of beamlets, and their relative weighting but was observed to be approximately 1.7%.

### 3.B Assessing DVH metrics

All box plots shown in the following sections were produced in MATLAB R2015b. The central mark displays the median value and the edges of the box show the 25th and 75th percentiles [the interquartile range (IQR)]. Outliers were identified as points >1.5 times the IQR above or below the boundaries of the IQR and are displayed as red crosses.

#### 3.B.1 Maximum plan dose

**Fig. 2:**
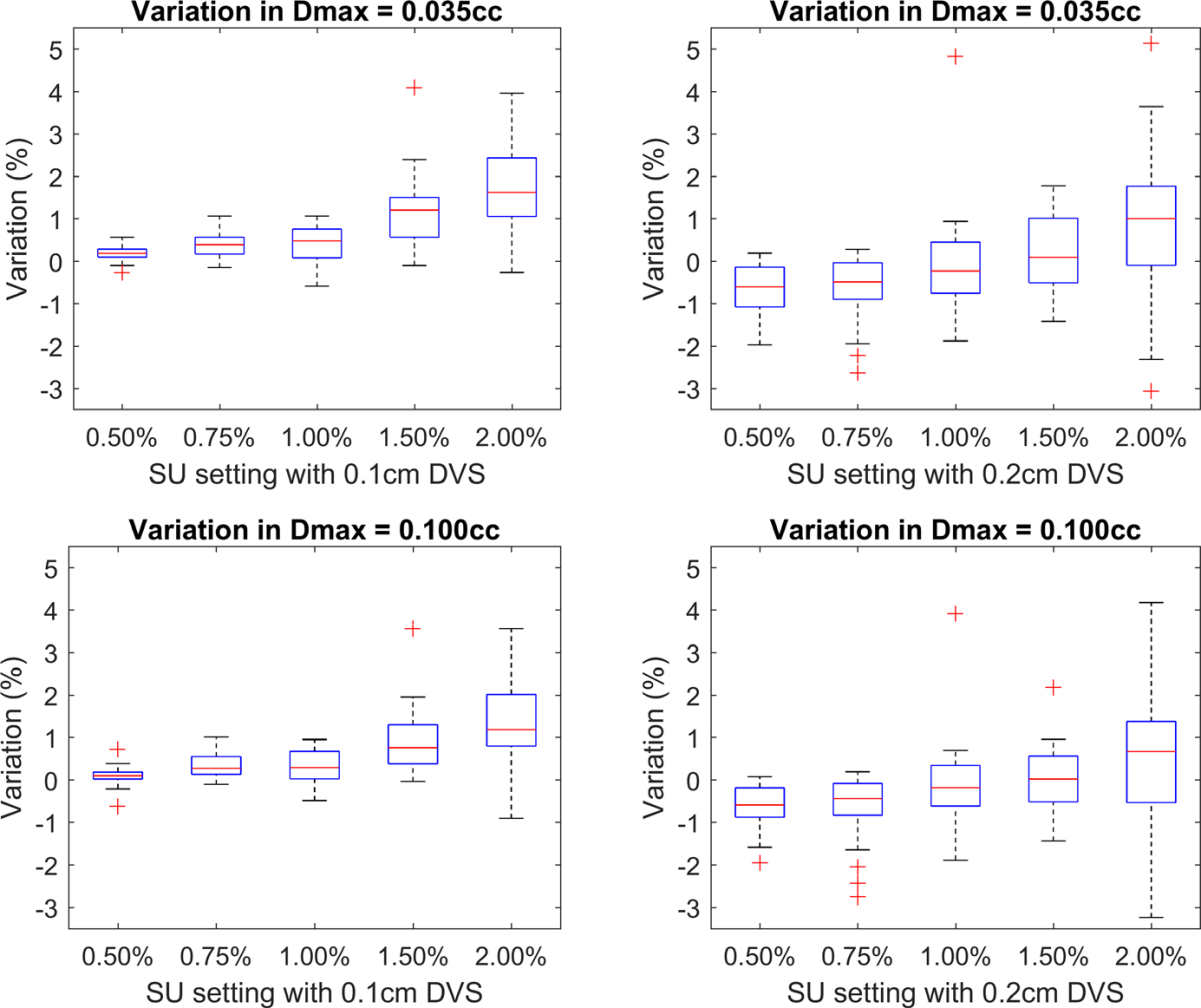
The top row shows the variation in maximum dose, defined as 0.035 cc, from the ground truth when calculated using a 0.1 cm (left) or 0.2 cm (right) dose voxel size (DVS). The bottom row shows the variations for maximum dose, defined as 0.100 cc, calculated using a 0.1 cm (left) or 0.2 cm (right) DVS.

Variations in the maximum plan dose within each calculated dose distribution are shown in Fig. 2 for all plans and for two definitions of maximum dose. The relative differences from the ground truth maximum dose are displayed due to the wide variations in absolute maximum dose between plans.

As the SU setting is increased, the reported maximum dose is seen to increase in all cases. Larger SU settings result in larger magnitudes of random noise being present in the calculation. By selecting the highest dose voxels from a distribution, those voxels with high doses combined with large positive noise values will be reported resulting in an artificial increase. This leads to an extension, or flick out, of the high dose end of the DVH curve.

When considering the calculations completed with a 0.2 cm DVS, the following observations can be made. For a maximum dose defined as 0.035 cc, the change in the median variation is greater between SU settings of 0.50% and 2.00% (1.6% change) than when defining the maximum dose as 0.100 cc (1.3% change). In addition, the IQRs are increased for a 0.035 cc definition of maximum dose compared to 0.100 cc. These results are duplicated for the calculations using a 0.1 cm DVS but to a lesser extent. A systematic reduction is also seen in the reported maximum dose compared to the 0.1 cm DVS calculations with the same SU. As a result, the calculations using a SU of 1.00% most closely represent the ground truth.

The changes in variation of the median and reduced IQR are due to the number of voxels included in the definition of the maximum dose. When using a 0.2 cm DVS, a maximum dose definition of 0.035 cc contains four to five voxels, whereas a definition of 0.100 cc contains 12–13. As a result, a single voxel with large random noise will have a larger effect on the 0.035 cc volume. For the 0.1 cm DVS setting, large values of random noise on a given voxel are less influential as a 0.035 cc contains 35 voxels. The systematic reduction in maximum dose is a result of an effective smoothing of the dose distribution due to the increased volume of each voxel.

The dose to a single voxel in a MC calculation should never be used during a plan assessment as some voxels will display doses multiple times the SU from their noise‐free value.^1^, ^4^ When defining the maximum dose, one must therefore compromise between using a number of voxels to reduce the influence of statistical noise and the associated volume of those voxels. If too few voxels are used, the reported maximum dose will be artificially high; however, if too many voxels are used, the reported value will correspond to a small volume rather than an approximation of a maximum point and may therefore result in an underestimation of the true maximum. For larger settings of DVS, fewer voxels are contained within a given volume and the user therefore becomes more susceptible to the effects of large random errors in a single voxel for a given volume of consideration. It is, however, important to note that for larger settings of DVS, the uncertainty per voxel will be reduced, resulting in the magnitude of random errors being reduced on average.

ICRU report 91 suggested recording the near maximum dose, defined as the dose to 0.035 cc for volumes < 2 cc to minimize errors associated with using a single voxel or small volumes.^4^ Based on the findings of this investigation, plan or target maximum doses defined as 0.035 cc, for calculations using a 0.1 cm DVS and SU of 1.50% or less, will report values within 1.5% of the ground truth value for 75% of the plans. Reducing the SU to 1.00% would reduce the variation to 0.8%. Small reductions in variation were observed when using a definition of 0.100 cc but were not adequate to compromise for the almost tripling of the volume. Calculations using a 0.2 cm DVS and SU of 1.50% or less reported values within 1.3% of the ground truth value for 75% of the plans. If higher levels of SU are used, however, a 0.100 cc definition of the maximum dose may be more appropriate for resilience to large statistical noise with calculations using a 2.00% SU showing <1.4% variation from the ground truth value for 75% of plans.

When considering the maximum dose to a specific OAR or target volume, it is also important to consider the native CT voxel size, the size of the volume, and the DVH calculation algorithm of the TPS.^24^, ^25^ When considering CT images with a fine resolution, or very small volume, the values suggested above may still be too large to represent the maximum dose. In these situations, the clinical team should consider reducing the SU to minimize the noise within the calculation and carefully review the local dose distribution rather than relying heavily on DVH metrics alone.

#### 3.B.2 PTV coverage

**Fig. 3:**
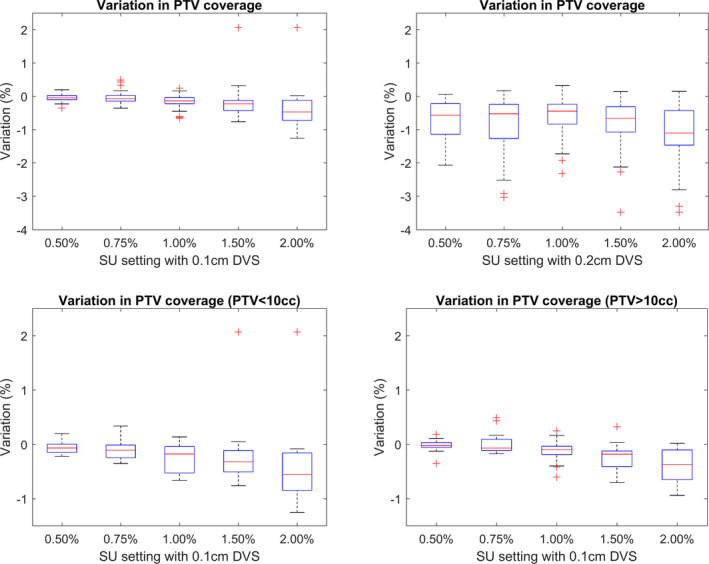
The top row shows the relative variation in planning target volume (PTV) coverage from the ground truth plan for calculations completed using a 0.1 cm (left) and 0.2 cm (right) dose voxel size (DVS). The bottom row shows only plans calculated with a 0.1 cm DVS split by PTV ≤ 10 cc (left) and PTV > 10cc (right).

The PTV coverage was defined as the dose received by 98% of the PTV for either brain or lung plans, and 90% of the PTV for spine plans. In the ground truth plans, the PTV coverage was achieved by the prescription dose. Figure 3 shows the relative variation in PTV coverage from the ground truth calculation.

As the SU value is increased, the reported target coverage is reduced. When considering all plans, calculations with a 0.1 and 0.2 cm DVS showed median variation decreases from 0.0% to −0.5% and −0.6% to 1.1%, respectively, for a change in SU of 0.5% to 2.0%. The calculations with a 0.2 cm DVS also show a systematically reduced coverage combined with larger IQR than the plans calculated with a 0.1 cm DVS. Those plans with a PTV volume of <10 cc (10 brain and two lung plans) showed a marginally steeper fall off in median target coverage and increased IQR when compared to plans with a PTV volume > 10 cc.

This reduction in coverage can be explained via the same logic as the increase in maximum dose described in Section 3.B.1. As the magnitude of the statistical noise is increased, minimum dose values within the PTV decrease as low dose values combine with large values of negative noise. The increased rate of reduction in coverage and increased IQR values associated with smaller PTV are a result of the reduced number of voxels. Large statistical noise values in only a few voxels have a greater effect on the reported values when the PTV consists of only a small number of voxels.

Similarly, systematic reduction in median PTV coverage and increased IQR values displayed for calculations using a DVS setting of 0.2 cm compared to 0.1 cm are both a result of the increased volume of a given voxel. For a PTV of fixed volume, the number of voxels associated with it decreases as the DVS is increased, resulting in larger variations in the volume receiving sufficient coverage due to statistical noise in any one voxel. In addition, the increased voxel size results in a larger volume averaging effect as dose delivered over a larger volume is attributed to a single location at the center of the voxel before subsequent interpolation.

When choosing optimal DVS and SU settings, it is important to remember the purpose of evaluation metrics. PTV coverage planning aims generally relate to achieving a required minimal dose. These reductions in reported PTV coverage with increasing SU or DVS will therefore not result in a plan being compliant with planning aims when the PTV coverage is lower than required. They may, however, result in an unnecessary increase in the planned PTV coverage which could lead to additional dose to OAR not needed to achieve treatment aims.

When using the highest tested SU setting of 2.00%, the PTV coverage was recorded above −0.7% of the ground truth plan, for 75% of plans when using a DVS of 0.1 cm, and −1.5% when using a 0.2 cm DVS.

#### 3.B.3 Spinal cord and PRV maximum dose

When producing SRT treatment plans for spine patients, the maximum dose to the spinal cord is of crucial importance and often determines the level of PTV coverage achievable as described in Section 2.E.

The variations in the maximum dose to the spinal cord or PRV follow a similar pattern to the maximum dose within the plan as shown in Fig. 2; however, larger IQR values are observed. The definition of 0.100 cc was chosen for the 0.2 cm DVS calculations for the reasons discussed in the Section 3.B.1.

Larger IQR values are a result of the localization of the spinal cord in a low dose area rarely within the primary fluence of the beam. Lower isodoses within the plan display an increased relative SU resulting in a greater variation in the reported doses as shown in Fig. 1. As the number of simulated histories is increased, the fraction of those which deposit dose in the spinal cord or PRV is significantly lower than those that contribute to the target region. As a consequence, lower SU settings are required to reduce the IQR associated with the OAR to those observed for the plan maximum doses.^2^

**Fig. 4:**
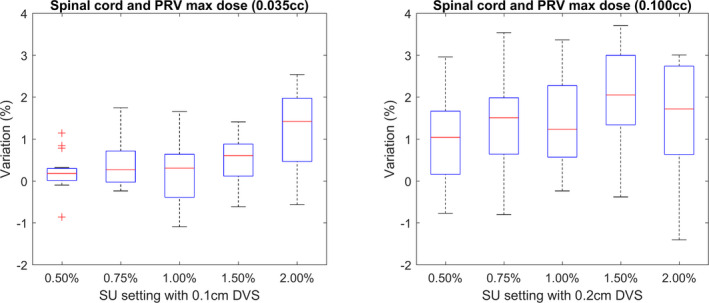
The relative change in the maximum dose reported to the spinal cord and spinal cord planning risk volume contours. The maximum dose is defined as 0.035 cc for the 0.1 cm dose voxel size (DVS) plans (left) and 0.100 cc for the 0.2 cm DVS plans (right).

As discussed with regard to PTV coverage, one should consider the purpose of the spinal cord or PRV maximum dose criteria. Plans are designed with the maximum dose to not exceed a given value to avoid associated treatment complications. As shown in Fig. 4, the statistical noise adds an additional safety net with a tendency to overestimate the dose. The use of a higher SU or DVS setting will therefore not generally lead to planning errors in which the calculated dose is falsely determined as planning constraint compliant. Conversely, meeting the constraint with an added safety net could lead to a reduction in target coverage as cord dose is prioritized over target coverage.^20^, ^21^, ^22^ Lower values of SU should therefore be considered to ensure precise reporting of the maximum dose in conjunction with the ability to achieve an optimal coverage of the target volume.

For calculations using a 0.1 cm DVS with a SU setting of up to 2.00%, the maximum reported dose did not vary by more than 2.0% from the ground truth value for 75% of plans. This was reduced to 0.9% by reducing the SU to 1.50%. When using a 0.2 cm DVS, a lower SU setting of 0.75% was required to maintain the reported maximum dose for 75% of plans within 2.0% of the ground truth value.

#### 3.B.4 GTV mean dose

Due to the steep dose gradients across SRT targets, the mean or median dose can be a more consistent way to report the delivered dose.^4^

**Fig. 5:**
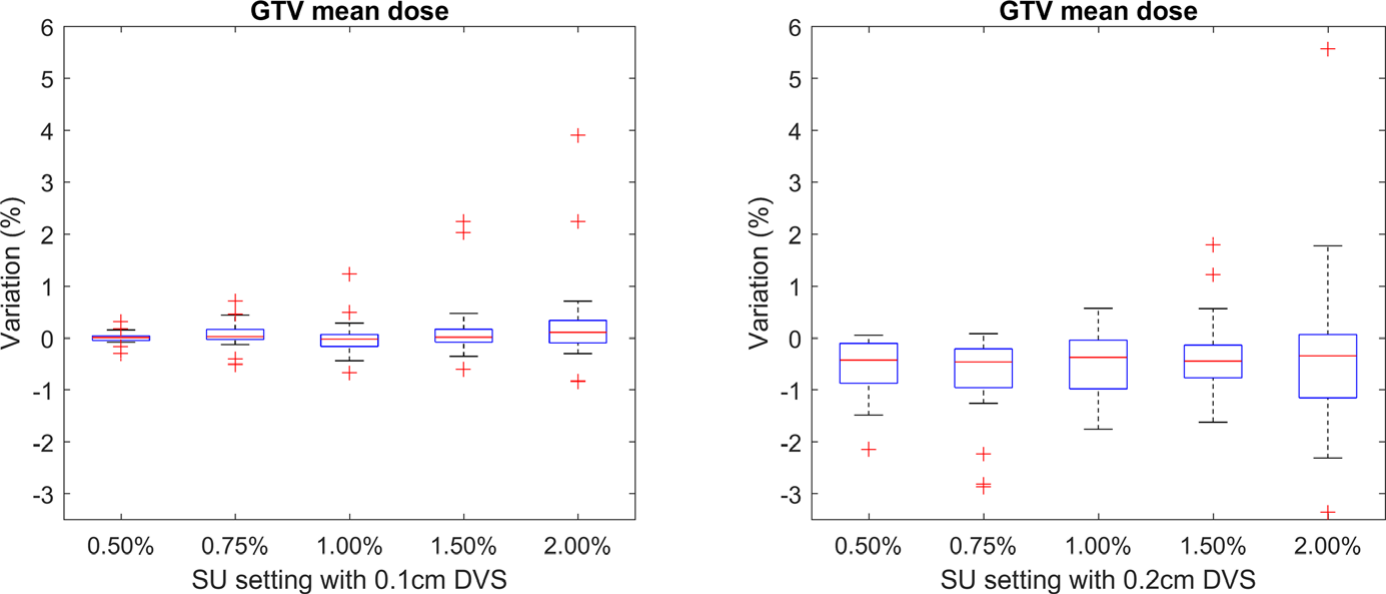
The relative change in the mean gross target volume dose from the ground truth for calculations completed using a 0.1 cm dose voxel size (DVS) (left) and 0.2 cm DVS (right).

The reported GTV mean dose did not vary systematically with increasing SU, and the IQR of the calculated values remained under 1.0% for all calculation settings, excluding the 0.2 cm DVS and 2.00% SU combination, as shown in Fig. 5. Due to the Gaussian distribution of the statistical noise, positive and negative noise values cancel out at every dose interval. Those plans which contained a particularly small GTV lead to outliers as a result of the low number of voxels considered. A systematic underestimation of the GTV mean dose relative to the ground truth plan calculated with a 0.1 cm DVS was observed again due to partial volume effects.

### 3.C Assessing plan quality indices

**Fig. 6:**
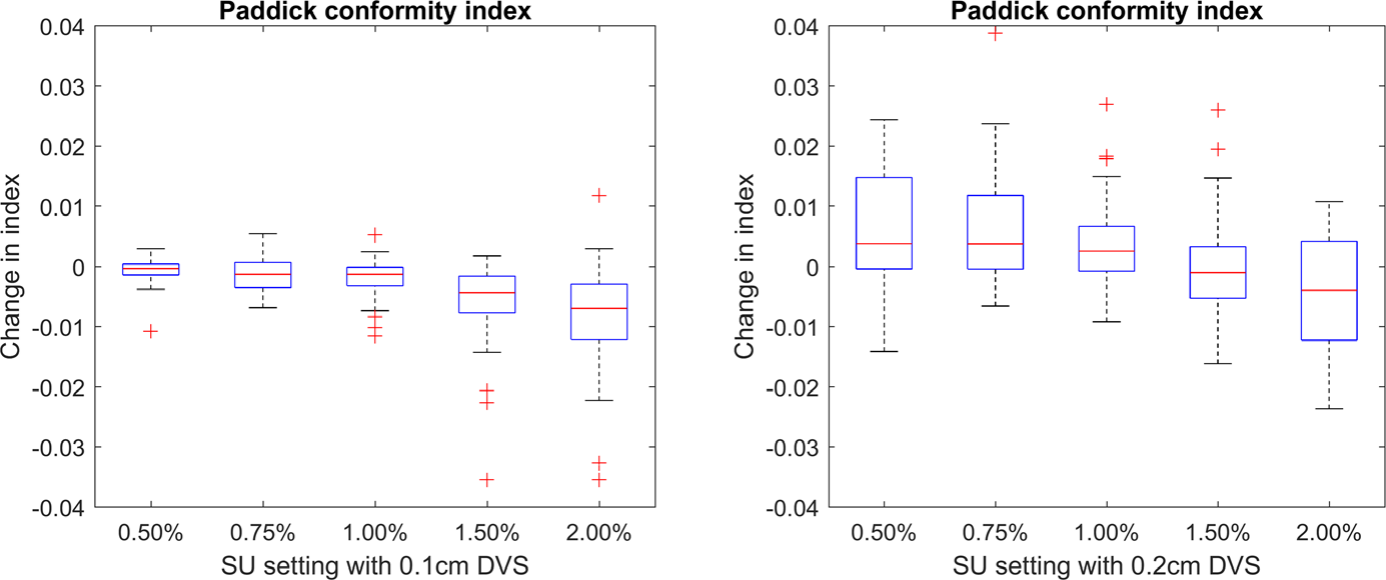
The absolute change in the Paddick conformity index from ground truth for calculations completed using a 0.1 cm dose voxel size (DVS) (left) and 0.2 cm DVS (right).

When assessing the effect of changing SU on the calculated PCI as described in Eq. (6), the predominant factor is the target volume covered by the prescription isodose line ($TV_{\mathrm{PIV}}$) which was shown in Fig. 3 to reduce with increasing SU. This leads to a reduction in the PCI, as shown in Fig. 6, as the target volume (TV) and the volume of the prescription isodose ($V_{\mathrm{RI}}$) remain approximately constant.

The GI index, as defined in Eq. (7), would only be expected to show a trend with respect to SU if the volumes of the prescription and 50% isodose line varied at different rates.

**Fig. 7:**
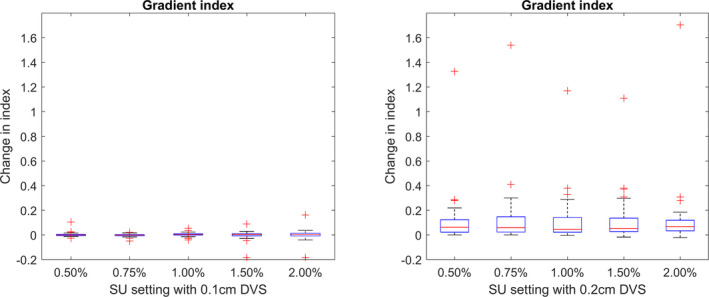
The absolute change in the Gradient Index from ground truth for calculations completed using a 0.1 cm dose voxel size (DVS) (left) and 0.2 cm DVS (right).

No overall trends were observed between the GI and SU settings as shown in Fig. 7. A larger offset was observed for calculations with a 0.2 cm DVS compared to the ground truth. It is important to note that these changes in plan quality indices are very small relative to the value of the index, typically in the range of 0.7 to 1.0 for the PCI, or 3 to 7 for the GI.

Due to the sensitivity of near minimum and maximum doses to targets and OAR to changing SU, it is desirable to also report on less sensitive metrics and indices such as the PCI and GI. From these results, it could be suggested that when planning with MC‐based systems, the roles of plan quality indices, such as the PCI or GI, can provide reliable information about a dose distribution for all settings of SU.

### 3.D Clinical implications

Throughout this study, a high resolution and high precision calculation has been considered a ground truth of the TPS and the underlying accuracy of the MC linac model has not been addressed directly. In clinical practice, importance should be placed upon agreement between the calculated and delivered dose distributions. If poor agreement is observed between calculation and measurements designed to verify the accuracy of the model, consideration should be given to possible improvements in the underlying MC model. During this process, it is important to maintain a good understanding of the uncertainties associated with the measurement and analysis processes, including the ability to falsely inflate gamma pass rates when high levels of statistical noise are present within a calculation.^26^

When considering the level of variation, which is acceptable, the clinical team should consider the uncertainty of the entire treatment process. The IAEA has recommended that systematic biases in radiotherapy should be <2%, and others, including the AAPM, have recommended that dose calculations in homogeneous media should be calculated within ±2% to ensure that doses can be delivered to a patient within ±5% of planned.^27^, ^28^, ^29^ While the increases in DVS and SU to a maximum 0.2 cm and 2.00%, respectively, have generally been shown to reduce the precision by less than 2%, this can constitute a reasonable fraction or the entire uncertainty budget.

### 3.E Treatment plan calculation time

The mean calculation times, subdivided into treatment area, for given DVS and SU settings are shown in Table 1. The area over which the dose calculation is completed cannot be controlled by the user but is instead defined by the volume of the data set and the associated structures. As such, calculation times can vary drastically between patients.

**Table 1:** The mean time taken to complete a calculation of a plan used within the investigation subdivided by treatment area. The values in brackets show the range of time taken.

Grid size (cm)	Statistical uncertainty (%)	Brain	Lung	Spine
Time to complete calculation (min)				
0.1	0.50	20.3 (7.0–38.0)	96.0 (61.5–220.0)	28.0 (14.5–60.0)
0.1	0.75	9.7 (3.8–17.0)	46.8 (22.3–92.0)	14.4 (8.3–33.0)
0.1	1.00	6.5 (2.5–11.3)	22.6 (9.3–43.0)	8.6 (6.0–13.0)
0.1	1.50	4.2 (1.5–7.0)	11.3 (5.0–22.5.0)	6.9 (3.5–13.0)
0.1	2.00	3.4 (1.2–8.0)	7.9 (2.0–17.3)	6.2 (2.8–11.8)
0.2	0.50	3.1 (1.0–5.8)	14.2 (5.0–48.0)	3.2 (2.3–6.5)
0.2	0.75	1.6 (0.8–3.5)	4.1 (2.0–8.0)	1.7 (1.4–3.0)
0.2	1.00	1.0 (0.5–2.3)	2.7 (1.3–5.8)	1.3 (0.8–2.0)
0.2	1.50	0.8 (0.5–1.5)	1.4 (0.8–3.5)	0.9 (0.5–1.3)
0.2	2.00	0.6 (0.4–1.0)	1.0 (0.5–2.5)	0.8 (0.4–1.3)

The lung plans took the longest time to calculate due to the large scan volumes which encapsulated the full lung volume. In contrast, the brain calculations were generally the quickest due to the small volume of the patient and treatment target over which the calculation was required. A calculation of approximately 10 min was expected to be practicable within the clinical environment and was achieved, on average, for the following settings.

- Brain plans with DVS of 0.2 cm and SU ≥ 0.50% or DVS of 0.1 cm and SU ≥ 0.75%
- Lung plans with DVS of 0.2 cm and SU ≥ 0.75% or DVS of 0.1 cm and SU ≥ 2.00%
- Spine plans with DVS of 0.2 cm and SU ≥ 0.50% or DVS of 0.1 cm and SU ≥ 1.00%

The variations between calculation times for varying DVS and SU settings were broad and not strongly correlated with the estimations which could be made using Eq. (3). This was a result of using a clinical system for which the performance was affected by the activity of other users, the 12% per control point limit placed on SU, and the predictive nature of Monaco in determining the number of histories required. For high SU plans, a greater than expected calculation time relative to a low SU setting was observed due to the 12% per control point limit which forced the achieved SU to be less than requested. For intermediate SU plans, the empirical formula used to determine the number of required histories can either under or overestimate the true value for the given plan depending upon factors such as the complex treatment plan, the number of control points, and target volume. The achieved SU can therefore be notably less than requested following the simulation of the calculated number of histories resulting in an increased calculation time. If an underestimation is made, additional histories are required and simulated before an assessment is again made of the achieved uncertainty ensuring no calculations are completed with an SU higher than requested and consequently no calculations are completed with a reduced calculation time. The result of this approach is that the average achieved SU across the cohort of treatments plans is lower than requested depending on the accuracy of the empirical formula in relation to the plans considered, as confirmed in Fig. 1, and the average calculation times are therefore difficult to predict based on relative changes in DVS and SU alone.

The calculation times relate only to the calculation of a plan dose distribution, and not the inverse planning process which would take significantly longer. To reduce the time required for the inverse planning process, the DVS or SU settings could be increased from the optimal values. These optimal values could then be used to complete a final calculation for DVH production. To minimize the differences between the plan generated by the optimizer and the final dose calculation, the quantified variations determined within this investigation could be used to update the optimizer aims. This approach is, however, limited by the maximum allowable SU value of 12% per control point, as discussed in Section 2.A, and one should be wary of the noise convergence error described by Jeraj.^10^

## 4 CONCLUSIONS

The specific settings required for calculation within a given clinical department should be considered by the multidisciplinary team, taking into consideration the resources available and the clinical requirements of the patient and treatment.

Throughout this investigation, it has been shown that changing the DVS setting has a greater effect on the displayed dose distribution than SU. As a result, reductions in the DVS setting should be prioritized over reductions in SU. In situations where a low DVS settings result in unacceptably long calculation times, a minimal increase should be made and considerations of intermittent values between 0.1 and 0.2 cm should be considered.

When reporting a DVH for a clinical plan, the DVS and SU settings used during the calculation should also be reported. The variations which have been quantified in this report could then be used to assess the expected ground truth DVH. Metrics and indices which have been shown to be resilient to statistical noise, such as the PCI, GI, and median doses, should always be considered alongside traditional metrics such as near minimum and maximum doses.

During evaluation of maximum or minimum doses, the definition should be carefully considered. The dose to 0.035cc is appropriate for plans calculated with a 0.1 cm DVS setting; however, a larger volume of 0.100cc might be more appropriate for plans calculated with a 0.2 cm DVS unless low values of SU are used to minimize the effects of statistical noise.

To generate treatment plans which can be calculated, on average, in under than 10 min, and for which the deviation of 75% of plans remains within 2.0% of the ground truth for the metrics considered here, the following SU and DVS settings are recommended.

- Brain plans with DVS of 0.1 cm and SU of 1.50%
- Lung plans with DVS of 0.2 cm and SU of 1.50%
- Spine plans with DVS of 0.1 cm and SU of 1.50%

In clinical practice, an additional safety margin on the choice of a 2.0% deviation and 75% of plans may be desirable. A reduction of the SU settings above to 1.00–1.25% could provide added confidence for a relatively small cost in time.

## ETHICAL STATEMENT

This study was approved by Cabrini Institute of Cabrini Health Limited, Victoria, Australia.
